# LONELINESS, ANXIETY AND DEPRESSION AMONG CAREGIVERS OF PERSONS WITH DEMENTIA: MEDIATING EFFECT OF SELF-EFFICACY

**DOI:** 10.1093/geroni/igad104.3454

**Published:** 2023-12-21

**Authors:** Alisha Thompson, Catherine Lemieux, Youn Kyoung (Lily) Kim, Laura Ainsworth, Rhonda Bardales, Julia Trahan, Scott Wilks

**Affiliations:** Louisiana State University, Baton Rouge, Louisiana, United States; Louisiana State University, Baton Rouge, Louisiana, United States; Louisiana State University, Baton Rouge, Louisiana, United States; Louisiana State University, Baton Rouge, Louisiana, United States; Louisiana State University, Baton Rouge, Louisiana, United States; Louisiana State University, Baton Rouge, Louisiana, United States; Louisiana State University, Baton Rouge, Louisiana, United States

## Abstract

A geriatrics enhancement program in southern United States piloted an interdisciplinary telehealth program to improve the health of caregivers of persons with dementia (PWD). Literature indicates that social isolation, loneliness, anxiety, and depression are common among PWD caregivers. When caregivers struggle to manage their own health, they may lose confidence in their health care abilities, i.e., caregiver self-efficacy. Using loneliness as the primary factor, the current study tested the mediating effect of caregiver self-efficacy on distinct outcomes of anxiety and depression. In a hospital setting in southern United States, the team in medicine, nursing, and social work collected data from 95 PWD caregivers on measures of loneliness, anxiety, depression, and caregiver self-efficacy. SEM with bootstrapping tested the two hypothesized mediation models, controlling for age, gender, and relationship to care recipient. The typical respondent was a 62-year-old Caucasian female providing care to a parent. Mediation model 1 direct effects were significant for loneliness on self-efficacy (β=-.4.24, p<.01); for self-efficacy on depression (β=-.02, p<.05); and for loneliness on depression (β=.67, p<.001). Indirect mediation effect of loneliness on depression through self-efficacy was significant (β=.09, bootstrapping 95% CI[.01, 21]). Model 2 direct effects were significant for loneliness on self-efficacy (β=-.4.19, p<.01), and for self-efficacy on anxiety (β=-.02, p<.05); direct effect of loneliness on anxiety was not significant (β=.04, p=.706). Indirect mediation effect of loneliness on anxiety was significant (β=.09, bootstrapping 95% CI[.02, 23]). Results underscore the importance of emphasizing social engagement, and including self-efficacy as a mediating factor, when treating caregivers’ anxiety and depression.

